# Higher Quality of Life and Lower Depression for People on ART in Uganda as Compared to a Community Control Group

**DOI:** 10.1371/journal.pone.0105154

**Published:** 2014-08-29

**Authors:** Faith Martin, Steve Russell, Janet Seeley

**Affiliations:** 1 School of International Development (DEV), University of East Anglia, Norwich, United Kingdom; 2 London School of Hygiene and Tropical Medicine, London, United Kingdom; 3 Uganda Research Unit on AIDS, Medical Research Council/Uganda Virus Research Institute, Entebbe, Uganda; Massachusetts General Hospital, United States of America

## Abstract

Provision of antiretroviral treatment (ART) to people living with HIV (PLWH) has increased globally. Research measuring whether ART restores subjective well-being to “normal” levels is lacking, particularly in resource limited settings. The study objectives are to compare quality of life and depression symptoms for PLWH on ART to a general community population and to explore factors to explain these differences, including socio-economic status and the impact of urban or rural residence. PLWH on ART (n = 263) were recruited from ART delivery sites and participants not on ART (n = 160) were recruited from communities in Wakiso District, Uganda. Participants were interviewed using the translated World Health Organisation Quality of Life brief measure, the Hopkins Symptom Checklist depression section, and questions about socio-economic status, residence as urban or rural and, for PLWH on ART, self-reported adherence and use of HIV counselling. Compared to the community sample and controlling for location of residence, PLWH on ART had significantly higher quality of life (QOL) for physical, psychological and environment domains, but not the social domain. These differences were not due to socio-economic status alone. Depression scores were significantly lower for PLWH on ART. Both comparisons controlled for the effect of location of residence. People on ART self-reported high adherence and the majority had used HIV counselling services. Our findings show better QOL amongst PLWH on ART compared to a general community sample, which cannot be explained solely by differences in socio-economic status nor location of residence. The general community sample results point towards the challenges of life in this setting. Access to health services may underpin this difference and further research should explore this finding, in addition to identification of psychological mechanisms that relate to better QOL. ART provision infrastructure has clear benefits. Further work should consider sustainability and replication for other health conditions.

## Introduction

The impact of antiretroviral therapy (ART) on the health of people living with HIV (PLWH) is well documented [1]–[4]. Over the last decade, the measurement of the effects of ART on quality of life (QOL) has been increasingly a focus of research, predominantly in industrialised countries [5]–[7]. As the roll-out of ART has increased in resource-limited settings, researchers have also begun to explore the impact of ART on QOL and more general well-being in those settings. A literature review of these studies show that PLWH on ART have significantly better physical health, emotional well-being, mental health and daily function compared to PLWH who are not on ART [1]. Similarly, ART is associated with lower depression compared to PLWH not on ART; as people's health and hope are restored, they can work again and engage socially [8]. However it remains unclear the extent to which PLWH on ART achieve similar well-being as people in the general community who are not on ART.

A better understanding of the well-being for PLWH on ART, compared to the general population, is important first as this provides an indicator of needs and intervention success. Identification of either low QOL or high depression may point to ongoing unmet needs. Second, achievement of good QOL over longer term may provide a motivator for ongoing adherence to ART [9], [10]. Finally, depression specifically is highly relevant owing to the global burden of the condition [11]. High depression may indicate a treatment need of high relevance to PLWH as depression is a barrier to adequate ART adherence [12].

QOL and depression are affected by a number of factors, including gender, age, religious beliefs and socio-economic status (SES) [13]. SES is more strongly associated to QOL in resource-limited settings [14], such as Uganda. SES may impact QOL through access to healthcare and fulfilment of basic needs [15]. The American Psychological Association draws researchers' attention to the importance of SES in relation to HIV/AIDS, advising considering SES particularly in research around well-being [16]. Differences in quality of life related to health may be mediated by SES: the impact of HIV on QOL may be explained by differences in SES associated with health status, such as inability to work owing to poor health.

While treatment can make HIV a manageable chronic condition [17] and may allow people to “resume relatively normal lives” [1], [9], there is limited information on the QOL and depression of PLWH on ART compared to a general community sample. Subjective QOL is not easily predicted by health status, as people may psychologically adapt to changes in health [18]. Additionally, in some resource-limited settings where substantial donor funding has been allocated to HIV services compared to other health services, it is possible that PLWH on ART have access to better counselling support and treatment than the general population [19], [20], as well as new sources of social support at clinics, such as meeting new people and involvement with HIV related community groups [21]. These resources may generate positive QOL effects compared to others in the general population.

ART could mean that PLWH continue to experience QOL below that of a general community sample, as they are still living with a serious condition and may experience long-term reduced health or side-effects [22], social exclusion or depression owing to losses experienced, and reduced life-expectancy [23]. Alternatively, ART could mean that PLWH experience QOL and depression at the same level as the general community sample, as their health may be returned to a more “normal” level with minimal side effects [24], health may mean a return of ability to work [25], signs of illness may be less visible meaning reduced concerns around stigma, and counselling and psychological adjustment may help maintain mood [9], [26]. Finally, ART could mean that PLWH experience higher QOL and less depression than the general community, as they may have access to new social support and health care services as a result of being HIV positive. Counselling or social support may provide new perspectives on life and skills that enhance well-being and psychological adjustment [9], [27]. This may be particularly relevant in a resource limited setting, where access to services and care may be limited for the general population. Both differing levels of resources and the effect of psychological adjustment and coping render it difficult to predict QOL in PLWH on ART.

Reviewing the evidence of the impact of ART on PLWH globally, a limited number of studies have compared QOL or depression between PLWH on ART and a general sample. Typically studies report higher depression in those with HIV than without [28], [29], although ART is linked to reduced depression in PLWH [24]. Lower QOL is reported in those with HIV compared to a general population sample, for example comparing PLWH on ART to a general sample in Vietnam [30] and in Malawi [31]. These studies include either people on ART for a short duration or made no selection based on ART duration. The reviewed research leaves questions of the impact of duration of treatment longer than 12 months unanswered. Notable exceptions to the pattern of worse outcomes in PLWH are recent findings from Uganda and South Africa, which showed greater subjective QOL and functional ability in older (aged over 50 years) PLWH on ART compared to those without HIV, explained in part by better access to healthcare for PLWH [20], [21], [32]. It is of interest whether these findings only apply to older adults.

The principal aim of this paper is to compare well-being for PLWH on ART to a general community sample in the resource-limited setting of Wakiso, Uganda. Specifically, subjective QOL and an indicator of depression are compared between PLWH who have been on ART for at least one year and a general community sample. In addition, the potential mediating role of SES is addressed.

## Methods

### Study site

Data were collected between January 2011 and March 2012 in Wakiso District, central Uganda. Three types of ART delivery sites in the District were selected to recruit participants: the HIV clinic at the government hospital in Entebbe; three government health centres outside Entebbe that have referral links to Entebbe; and the Entebbe branch of a well-established non-governmental organisation, The AIDS Support Organisation (TASO).

Wakiso District, which encircles the capital city Kampala, was selected as the site for ease of access to the study population (the research project and team were based at the Medical Research Council/Uganda Virus Research Institute [MRC/UVRI], Entebbe) and because of the presence of established government and non-government treatment providers. This region includes both more urban and more rural communities; however, this distinction is not a simple one as there is substantial peri-urban development and people may travel between communities.

### Sample

For the PLWH on ART sample, participants must have been on ART for more than one year to be eligible. A list of eligible patients was compiled for each facility, which was then stratified by age and gender. The sample was selected randomly from the ART delivery sites using systematic random sampling intervals. The participants from the general population were randomly recruited from nearby villages, first stratified by gender, using resident lists from communities which had been part of an earlier unrelated MRC/UVRI study. It must be noted that HIV status was not determined in the control sample. The data collected herein are part of a wider study “Life on antiretroviral therapy: People's adaptive coping and adjustment to living with HIV as a chronic condition in Wakiso District, Uganda”, funded by the UK Economic and Social Research council (grant number RES-062-23-2663). Sampling for the control group was completed using an existing list of residents in several communities held by the Medical Research Council from previous studies. Sampling was not stratified by age nor by urban vs rural variations.

### Data collection measures

Measures assessed quality of life, mood and socio-economic status. In addition, participants provided information about location of residence as urban or rural, their age, gender, household size, years of education, religion, marital status, use of medication and HIV counselling and self-reported adherence over the last three days and if they had missed pills more than three times in the last month [33].

The fifteen item depression section of the Hopkins Symptom Check List (DHSCL) [34] was used. Scores range from 15 to 60, with higher scores indicating more symptoms of depression. This measure is commonly used as a screening tool for depression and has been validated for use in a range of cultures [35]–[37]. It is validated for use in Uganda in the Luganda language and has good predictive validity to detect depression and good internal consistency [38]. The measure has a cut-off score to indicate likely presence of depression. The Hopkins checklist has been researched to identify a cut-off point that indicates the likely presence of depression. This cut-off point has been found to be related to context, culture and measurement version. For example a mean score greater than 1.75 is used in Norway [39] and 1.03 for pregnant women with HIV in Tanzania [40]. Studies using the Ugandan version of the measure (as used in this study) have validated and used a cut-off of above 30 (or an individual's mean score of 2) to indicate probable depression [41], [42]. This same method is used here to allow comparisons to other studies in Uganda.

To assess quality of life, the World Health Organisation (WHO) Quality of Life measure the WHOQOL-BREF was used [43]. Twenty-six items assess four domains of quality of life: 1) physical health, 2) psychological, 3) social relationships and 4) environment. Higher scores indicate better quality of life. Domain scores range from 4–20. This measure was selected as it provides an estimate of quality of life extending beyond health-related quality of life alone and has been shown to be valid cross-culturally [43]. The measure was translated into Luganda using the WHO guidelines and achieved adequate validity and internal consistency, comparable to other translated versions [44].

Participants were also asked to complete items forming a socio-economic status (SES) score. SES scores were created from variables of owning one's home, having electricity, having piped water, having a good toilet (flush), having a house with a good roof (iron/tile) and having a paid job (skilled manual labour, formal business, professional or service job). The SES scores were created through inverse frequency weighting, where rarest items are given greater weighting in the score (weights for each variable are derived by dividing total sample size by number of participants who have the item, these are then summed to create the overall score). Higher scores indicate greater assets and socio-economic status.

### Ethical considerations

Ethical approval for the study was obtained from the Uganda Virus Research Institute and the University of East Anglia, UK. Overall approval was granted by the Uganda National Council for Science and Technology. Informed consent was gained from participants in written format, or witnessed oral consent was used, endorsed by thumb print. These consent procedures were ethically approved.

### Analysis

Statistical data were analysed using PASW Statistics SPSS version 18. Differences between the characteristics of the HIV and control group were compared, using t-tests or the appropriate non-parametric equivalent (Mann-Whitney U). ANCOVA was used to compare depression and QOL scores between the groups. ANCOVA was chosen to covary for any variables where there were significant differences between the groups' characteristics. ANCOVA was only conducted after checking for homogeneity of regression for the covariate at each level of the independent variable. Homogenity of regression was assessed by calculating the interaction terms for the independent variable and covariate. Effect sizes for differences in relation to HIV group were calculated using a standard method to include consideration of the variance of the covariate [45].

Correlation analysis was performed using Pearson's r, as data were found to be normally distributed, and was used to explore the relationship between variables. Group sizes were unequal comparing the HIV group and control group; therefore, corrections for unequal variances are reported where necessary (reflected in the reported degrees of freedom). Effect sizes are included (Cohen's *d*) where appropriate.

Mediation analysis was used to test the significance of the indirect effects of the independent variable or “IV” (HIV group or control group) on the dependent variables or “DV” (WHOQOL domains) through a potential mediator or M (socio-economic status). Mediation analysis addresses the degree to which differences between the groups' QOL may be due to the impact of SES. The mediated or indirect effect is the product of effect of IV on M (*a*) and the effect of M on the DV (*b*), partialling out the effects of the IV on the DV [46]. The direct effect (*c′*) is the relationship between IV and DV, taking account of the mediated effect. The total effect (*c*) is the total relationship between IV and DV. Mediation analysis used bootstrapping to estimate the indirect (or mediated effect) size, which were considered significant when the 95% confidence interval did not include zero [47], [48]. The SPSS Macro was used to calculate the model components [49]. There is no agreed upon way of calculating how much of the variables effect is due to mediation [50]. The method used here is to calculate the proportion of the total effect that is mediated (creating a percentage by dividing indirect effect by total effect, subtracting this from one and multiplying by 100).

## Results

### Demographic differences between HIV and control group

Table 1 shows the comparison between demographic data for the PLWH on ART group or “HIV group” (n = 263) and control group (n = 160). Prior to exploring any differences in outcomes of depression and quality of life scores between the groups, it was essential to ensure that the groups were comparable in terms of other variables that may have an impact on these outcomes. Comparisons of marital status (χ^2^(2) = 5.24 p = 0.07), gender (χ^2^(1) = 3.16 p = 0.076), religion (χ^2^(1) = 0.21 p = 0.65), education level (χ^2^(2) = 1.23 p = 0.54) and age (t(239.5, equal variances not assumed) = 0.016 p = 0.99) revealed no significant differences between HIV and control group. As such, there is no requirement to control for these variables when comparing QOL or depression scores for the two groups.

**Table 1:** Demographic details of the HIV and Control groups.

Variable	HIV (n = 263)	CONTROL (n = 160)	p value
Mean Age in years (s.d.)	39.8 (9.76)	39.8 (15.22)	0.99
Mean number of people living in household (s.d.)	4.6 (2.68)	5.3 (3.33)	0.022
Socio-economic status (s.d.)	6.4 (4.32)	5.3 (3.96)	0.01
	Frequency (%)	Frequency (%)	
Urban	113	19	<0.0001
Rural	150	141	
Number female	177 (67.3)	94 (58.8)	0.076
Education level			
None	32 (12.2)	17 (10.6)	0.54
Primary	144 (54.8)	93 (58.1)	
Junior	3 (1.1)	4 (2.5)	
Senior school	62 (27.3)	40 (25.0)	
Further education	12 (4.6)	6 (3.8)	
Religion			
Christian	226 (85.9)	140 (87.5)	0.65
Muslim	37 (14.1)	20 (12.5)	
Marital status			
Single	60 (22.8)	27 (16.9)	0.07
Married	155 (58.9)	112 (70.0)	
Other	48 (18.3)	21 (13.1)	

Total household size was significantly different (t(283) = 2.309, p = 0.022, mean difference 0.72, 95% CI 0.10–1.33). When household size was correlated with WHOQOL scores and depression scores, only correlation with the WHOQOL psychological domain (domain 2) was significant (r = −0.1, p = 0.042). This was a weak correlation. There were significant differences in urban compared to rural residences of the two groups, although this division is a simplification as there is peri-urban development. Those in the HIV group were more likely to be living in urban regions than those in the control (χ^2^(1) = 43.357, p<0.0001). As such, comparisons between HIV group and depression and WHOQOL outcomes included location of residence as a covariate.

Socio-economic status (SES) was significantly different between the two groups (t(421) = 2.589, p = 0.01). SES was significantly higher for people with HIV, although this was a small difference (effect size *d* = 0.26). SES correlated significantly with depression (r = −0.11, p = 0.031). The correlations between SES and physical health (WHOQOL domain 1, r = 0.10, p = 0.035), WHOQOL psychological (domain 2, r = 0.14, p = 0.003) and environment (WHOQOL domain 4, r = 0.20, p<0.001) were significant, although only demonstrating a weak relationship. Mediation analysis was conducted to take into account the impact of SES on group differences for the WHOQOL domains and depression symptoms. Although social relationships (WHOQOL domain 3) did not correlate significantly with SES (r = 0.09, p = 0.052), mediation analysis was conducted for completion.

### ART and HIV counselling use in HIV group

All 263 participants in the HIV group were taking ART at the time of the study, having started ART more than one year prior to the start of the study. Of the HIV group, 250/263 (95.1%) had received HIV counselling. Self-reported adherence revealed 19 participants (7.2%) reported missed pills in the last 3 days and 12 (4.6%) reported having missed their pills more than three times in the last month.

### Comparing depression between HIV and control group

Mean scores on the DHSCL were calculated by group. The HIV group's mean score was 22.0 (s.d. 5.50), compared to 24.9 (s.d. 6.44) for the control group. The mean difference is −2.92 (95% CI −4.17–−1.67), with higher scores in the control group, indicating more symptoms of depression. There was a significant difference between the two groups for depression scores after controlling for the effect of location of residence (F(1,393) = 4.315, p = 0.038). The effect of the covariate was also significant (F(1,393) = 4.076, p = 0.044). Taking account of the covariate variance, the effect size for HIV group was 0.50 (95% CI 0.29–0.71).

Chi-square test revealed that depression and group were associated, as χ^2^ (df-1) = 10.82, p = 0.001, phi = 0.161 (small effect size). The results in Table 2 illustrate how proportionately more people in the control group are potentially depressed.

**Table 2:** Indicated depression by HIV or control group.

Depression indicated	HIV group (%)	Control group (%)
Yes	24 (9.6)	31 (21.1)
No	226 (90.4)	116 (78.9)

### Comparing Quality of Life between HIV and control group

Table 3 provides mean scores on WHOQOL domains by HIV and control group. ANCOVA revealed that scores were significantly higher in the HIV group physical health (WHOQOL domain 1), after controlling for the effect of location of residence (F(1,419) = 5.123,p = 0.024). The effect size, taking into account the covariate, was medium (d = 0.43, 95%CI 0.23–0.63). The covariate was not significantly related to physical health QOL (F(1,419) = 3.486,p = 0.062).

**Table 3:** WHOQOL Scores by HIV and Control group.

WHOQOL Domain	HIV group mean (s.d.)	Control group mean (s.d.)	Mean difference	95% CI of mean difference	Effect size (d)
1 Physical health	14.4 (2.15)	13.3 (3.17)	1.09*	0.54–1.65	0.43
2 Psychological	16.6 (1.80)	14.9 (2.52)	1.69*	1.24–2.13	0.80
3 Social relationships	14.6 (2.75)	14.0 (3.16)	0.65 (n.s.)	0.08–1.23	-
4 Environment	14.2 (2.04)	12.4 (2.74)	1.74**	1.24–2.23	0.75

** indicates significantly different at p<0.01,

* indicates significance at p<0.05, based on ANCOVA results. Reported effect sizes take into account the variance relating to the covariate (location of residence).

For domain 2 (psychological) ANCOVA revealed significantly higher scores for the HIV group after controlling for the effect of location of residence (F(1,419) = 6.591,p = 0.011). The effect size, taking into account the covariate, was large (d = 0.80, 95%CI 0.60–1.00). The covariate was not significantly related to psychological QOL (F(1,419) = 1.765,p = 0.185).

For domain 3, social relationships, ANCOVA revealed no significant differences between the groups after controlling for the effect of location of residence (F(1,419) = 0.73,p = 0.786). The covariate was not significantly related to social relationship QOL (F(1,419) = 2.874,p = 0.091).

For domain 4, environment, ANCOVA revealed significantly higher scores for the HIV group after controlling for the effect of location of residence (F(1,419) = 14.518,p<0.001). The effect size, taking into account the covariate, was medium (d = 0.75, 95%CI 0.55–0.95). The covariate was not significantly related to environment QOL (F(1,419) = 2.550,p = 0.111).

### Mediation analysis: HIV group and WHOQOL in relation to SES

Mediation analysis was completed to explore the degree to which SES is affecting the relationship between case and quality of life or depression symptoms. In all cases, the effect of HIV group (independent variable) on the mediator SES was 1.0869. Table 4 provides the summary analysis.

**Table 4:** Summary of Mediation analysis of socio-economic status and the relationship between HIV case and WHOQOL domains (Unstandardised co-efficients are shown).

Dependent variable	Direct effect of HIV group on DV (*c′*)	Indirect effect (*ab*)	95% CI of indirect effect	Total effect (*c*)	Proportion %
Depression	2.7895**	0.1301	−0.3882–0.008	2.9196**	4.45609
WHOQOL 1 (Physical health)	1.0404**	0.0534	−0.0030–0.1698	1.094**	4.89945
WHOQOL 2 (Psychological)	1.6267**	0.0587*	0.0078–0.1607	1.685**	3.45994
WHOQOL 3 (Social relationships)	0.5903*	0.0619	−0.0016–0.1963	0.6522*	9.49095
WHOQOL 4 (Environment)	1.6338**	0.1018*	0.0241–0.2316	1.736**	5.88710

* significant at p<0.05.

**significant at p<0.01 Proportion calculated as (1−(c′/c))*100.

For all comparisons, the relationship between HIV group (case or control) and the WHOQOL domain or depression remained significant when the effect of SES was accounted for: there is no full mediation of HIV group and QOL or depression by SES. There was only partial mediation by SES for WHOQOL 2 (psychological) and 4 (environment) domains. However, the effect of SES on the HIV group to QOL relationship was very small. The proportion of the total effect that was mediated was calculated (Table 4). This illustrates the minimal proportion of the relationship between HIV group and QOL that was found to be mediated by SES, with between 3.6 and 5.9% of the relationship between IV and DV mediated by SES.

## Discussion

Compared to a general population sample, greater QOL was seen for PLWH on ART for physical, psychological and environment domains after controlling for the effect of location of residence. Social relationship (WHOQOL domain 3) scores were not significantly different in relation to group. Although socio-economic status was higher in the PLWH on ART, this accounted for at most 6% of variance in QOL scores. Mean depression scores (after controlling for the effect of location of residence) and number of people reaching a threshold for possible depression diagnosis were lower for PLWH on ART. PLWH on ART were found to achieve better well-being than the general community sample.

At first glance these results seem counter-intuitive: that people with a chronic, incurable illness should have better QOL and lower symptoms of depression than the general sample. One possibility is that these results may tell us about the lives of the general sample in Uganda. Many people live in harsh economic and social conditions, and experience living costs that outstrip income [51], and gender inequalities can make women's lives and relationships particularly difficult [26]. Access to affordable essential services remains a problem for many and PLWH on ART appear to have better access to these services.

The HIV sample reported generally high levels of adherence. Self-reported adherence was used, and this may be an inaccurate estimate [52]. Most of the HIV sample had received HIV counselling (although the quality of this is unknown). Other studies show high adherence levels on ART, where available, in sub-Saharan African settings [53], [54]. The QOL and depression results presented here are therefore for a sample of people who appear to be adhering to ART and using services. A different pattern of results may be observed comparing PLWH on ART with lower adherence or with lower access to counselling services. Adherence to ART and the impact of ART on QOL and depression may change as the length of time a person is taking ART increases [55]. Longitudinal studies should explore how QOL and depression may alter over time and taking into account levels of adherence.

This study is one of the first studies in sub-Saharan Africa to provide a direct comparison between an HIV and non-HIV group, showing the rates of depression for the general population are higher. QOL scores, if viewed as a percentage of scale maximum, ranged across the domains from 71% to 83% for PLWH on ART and 62% to 74.5% for the general sample. These ranges are similar to findings that QOL among non-western populations is generally around 60–80% of scale maximum [56]. Although there were significant differences in depression scores overall, the two groups' mean depression scores were not clinically different (the means for both groups were below the clinical cut-off). However, we found significantly more people scoring above the cut-off for likely depression in the general sample as compared to the PLWH on ART group. Typically studies find higher depression in PLWH compared to the general population; however, the findings here may reflect the positive impact of ART and counselling. Twenty-one percent of participants in the control sample reached DHSCL scores indicating potential depression, compared to only 9.6% of the HIV sample. Previous research found similar rates of depression symptoms with a national prevalence in Uganda of 29.3% [42] and 8.1% in an HIV sample [41]. As the rate of attendance at HIV counselling was very high in our sample, it was not possible to explore any effect of counselling on reported mood.

PLWH were receiving counselling, care and treatment from specialised HIV services provided by the state and non-governmental organisations, notably TASO. HIV service infrastructure has received a great deal of investment since 2004/5, substantively enhanced by donor funding. These services may impact QOL domains, including offering access to health care (environment domain) and benefits of health care (physical). In contrast the sample from the general population are unlikely to have been accessing specialised treatment and counselling services.

Inequalities of service provision for PLWH compared to other conditions or the general population have been found elsewhere [57], and a study in South Africa recently found greater QOL in older adults with HIV compared to those without HIV, explained by better access to healthcare for PLWH [20]. ART facilities provide something akin to “islands of excellence in seas of under provision” [58]. Individuals in both the case and control groups live in difficult circumstances and this study did not collect detailed data regarding service access. However, through contact with ART clinics, PLWH had access to a service which gave them the opportunity to talk about their problems in counselling sessions or with their in-group of PLWH. This may relate to the higher reported QOL and should be investigated in further research.

Research exploring psychological adjustment and the concept of “post-traumatic growth” suggests that following health shocks and/or treatment, people may experience a renewed sense of well-being [59]. Gratitude for a new chance at life, a refreshed sense of priorities and a shift in standards against which QOL is judged can all lead to increased subjective QOL following illness [60]. Studies with PLWH in various settings, including Uganda, have found descriptions of this renewed sense of meaning and gratitude [61], [62]. It may be that these adjustment processes underpin observed differences, together with different access to resources.

It must be noted that not all PLWH on ART experience good QOL. Identification of people with high reported QOL could be used as peer counsellors to support those who are faring less well on ART or could be used to help motivate those who are refusing/struggling with adherence to ART. The lower QOL and lower mood in the community sample also suggests a need for provision of psychosocial intervention more widely. Greater understanding of the factors contributing to outcomes in the general community is required to help target any such intervention and explore wider policy development regarding improvement of well-being.

### Limitations

This study has several limitations. Although not statistically significant, there were more women and single people in the PLWH on ART group. Further research could explore the relative relevance of these variables to well-being outcomes.

ANCOVA was conducted to take into account the covariate of location of residence. The urban-rural dichotomy does not take into account the many differences between the sites, where development initiatives have led to the provision of services in the rural environments and infrastructure that may be more similar to a peri-urban environment. Additionally, although location of residence may be in a more urban area for example, work may take place in a more rural environment or vice versa. As such, a more detailed examination of the impact of participants' environment would be of benefit.

Although SES was measured with a scale specifically designed for this population, SES is difficult to measure. The measure here included assets and employment items. Alternatives such as a longer assets index or measure of expenditure versus income may have revealed different results.

Self-reported adherence was used to estimate adherence. Adherence is difficult to measure accurately and alternative, objective or more detailed measures of adherence should be included in future research. In addition, data relating to counselling only concerned attendance at counselling. Further research should consider the quality and content of this intervention to properly understand its effect on QOL and depression.

It is probable that some of the general population sample are HIV positive and untreated, because HIV prevalence is estimated at 7.3% [63] and many people are un-tested. It was not possible to account for this in the data analysis. No data were collected around objective health measures or types of treatment, therefore it is not possible to know the extent to which differences in health QOL were due to better health in the ART group. It is well known that although people with poor health typically have lower QOL than those without health problems [43], the relationship between health status and QOL is complex, particularly in relation to chronic conditions [64]. Further research should explore the relative roles of objective health indicators and psychological adjustment in relation to QOL judgements. The generalizability of findings is unclear, although our results add to an emerging evidence base in sub-Saharan African nations were HIV treatment is accessible that shows better well-being amongst PLWH (on ART) compared to a general sample [20], [21], [32].

## Conclusions

PLWH on ART were experiencing higher QOL and lower depression scores than the general community sample. The precise mechanisms driving these results need to be investigated. It may be that PLWH on ART experience better health than the control group, particularly as this sample reported good adherence. PLWH on ART may have better access to healthcare resources than the general community and may receive not only ART but also treatment for other illness, for example, access to antibacterial medications which treat common infections. As such, access to ART may also provide better access to healthcare overall. Further research could directly explore and measure these mechanisms to quantify their impact on QOL and depression. The needs of the general community sample and factors associated with their lower QOL also require further research and potential intervention.

As HIV care is rolled out to lower level health centres in Uganda and elsewhere and gradually integrated into the general health system, it is useful to reflect on the particular benefits that PLHW in our study may have derived from the infrastructure that has been put in place because of HIV. People with other chronic conditions, such as hypertension, might also benefit from such regular social and medical support, but in a financially resourced constrained world the provision of such services may decline, with consequences for ART adherence as well as the QOL of PLHV.
